# An Elementary and Practical Treatise on Internal Pathology

**Published:** 1847-07-01

**Authors:** 


TRAITÉ ELÉMENTAIRE ET PRATIQUE DE PATHOLOGIE INTERNE. Par *A. Grisolle*, M.D.P., Médecin de l'Hôpital St. Antoine agrégé à la Faculté de Médecine. Deuxieme Edition, revue et augmentée.

AN ELEMENTARY AND PRACTICAL TREATISE ON INTERNAL PATHOLOGY. By *A. Grisolle*, M.D. P. Second Edition. In Two Vols. Octavo, pp. 1679.

A SYSTEMATIC work on Internal Pathology, or, as we should call it in this country, on the practice of medicine, from the pen of a distinguished physician of one of the Parisian hospitals, and which is already before the public in a second edition, cannot fail to claim the attention of the British practitioner and medical student. But its importance becomes more apparent when it is known that the views which the author sets forth have been principally taken in the wards of La Charité, and from the practice of Professor Chomel, who may be justly regarded as one of the most eminent, eclectic, or Hippocratic physicians of the present day. In this respect the author has trodden in the footsteps of Professor Louis, and may be esteemed of the same medical school with him and Professor Andral.

It is the design of the work to exhibit the state of medicine at the present day, as it exists not in France only, but in the other countries of Europe, and in the United States. But, although the author has shewn a better acquaintance with English medical writers than is often to be found amongst French physicians, it is evident that even he is imperfectly informed regarding the state of medicine in England.

In a systematic work like that of Dr. Grisolle the arrangement of the subject is a matter of no small importance. The author has adopted that which he calls a philosophical or nosological system, by which it is to be understood that his object is to bring together those diseases which are allied by the character and nature of the symptoms which they present, and which may therefore be supposed to require a parity of reasoning for their explanation, and analogous treatment for their cure. He prefers this to a pathological system, which might be founded either upon the classification of tissues, in which the particular derangements characteristic of different diseases occur, or upon the systems of organs subservient to the different functions of the body. There are advantages presented by each of these methods, but in the opinion of the author they are not equal to those of a nosological arrangement. The alphabetical order he rejects at once, condemning it as the worst that could be selected. In this opinion, if it is to be restricted to a comprehensive work for the use of the medical student, there is no one acquainted with the subject who will not readily concur. But in the application of the alphabetical method, as practically employed, it must be remembered that it is almost entirely restricted to large works of reference, such as the various Encyclopædias of medicine and surgery which are adapted to the use of those who are engaged in practice, or who may be already far advanced in the study of their profession. Each article in such works constitutes a monograph, in which the attention of the writer, as well as that of the reader, is concentrated upon the particular subject without his being necessarily obliged to consider what may be advanced in other parts of the extended work. In such a publication, the superiority of an alphabetical arrangement cannot be disputed.

The author observes with Requin, that Nosography must, in the present day at least, be neither exclusively organic, ætiological, nor symptomatic, but must assume a triple character. A mixed system must therefore be adopted to avoid mutilating the science, or leading it astray into hazardous hypotheses.

On this principle the following division into ten classes is employed:—

I. Fevers.
II. Diseases consisting in a fault in the proportion of blood.
III. Inflammations.
IV. Hæmorrhages.
V. Morbid secretions.
VI. The results of poisons.
VII. Diseases of nutrition.
VIII. Structural changes and accidental productions.
IX. Nervous diseases.
X. Diseases peculiar to certain organs or tissues.

We shall not attempt to criticise this classification, which the author does not pretend to be perfect. The number of authors who have already tried their hands on the construction of nosological systems sufficiently attest the difficulty of the task. These systems, which are necessarily more or less artificial, serve as the frame-work or skeleton to the body of facts which are accumulated, and provided the principle be adhered to by the author, and comprehended by the reader, the principal object will be attained. It is of more consequence, as Dr. Grisolle remarks, to be accurate in the special details, and to these he has carefully, and we believe successfully, applied himself. As he informs us in the conclusion of his preface, he has treated of special Pathology without attempting those views which belong to general Pathology and Semeiology, for which he refers to the classical work of Professor Chomel, which he regards as unquestionably the most remarkable introduction to the study of medicine which has yet been produced.

### FEVERS.

In making a distinct class of fevers, the author necessarily rejects the views of those who have endeavoured to reduce all these diseases to the class of inflammations, and says—" I believe there is no one, in the present day, who would dare to defend such a doctrine. On the contrary, every one admits the existence of diseases of which fever forms the predominant character, and which are not connected with any local derangement; or in which, if the solids are affected, their lesion is subsequent to the fever, and incapable of explaining it, being, like the fever, the effect and consequence of a more general cause."

He proceeds to enumerate the general phenomena of fever, and speaks of it as an element to be considered in forming a diagnosis. He repeats the remark of Andral, that in fevers not attributable to any local derangement there is everywhere a tendency to inflammatory action, which, if the fever continue, will give rise to various phlegmasia according to individual predisposition, or to the varied susceptibility of particular organs. Piorry believes that, in these cases, there exists a primitive inflammation of the blood, to which he gives the name of *hémite*. The author divides fevers into—

1. Continued fever—including ephemeral, inflammatory fever—typhoid fever, (the typhus of Europe)—typhus fever of the English—the bilious fever of warm climates—yellow fever, and the typhus of the East.
2. Eruptive fevers, such as small-pox, chicken-pox, measles, scarlatina.
3. Intermittent, containing the mild, pernicious, and anomalous.
4. Remittent and pseudo-continued fevers, which are rather to be regarded as a sub-genus of the intermittent.
5. Hectic, lingering, or chronic fevers.

Under the head of "typhoid fever" the author treats of that important febrile affection which constitutes the greater number of severe cases of fever which occur in our temperate zone, and which has received a variety of appellations, of which the following are enumerated at the head of the article:—Phrenitis, of the Greeks and Latins; Pestilential, Malignant, Putrid, Bilious, Mucous Fevers, of most authors; the low nervous Fever of Willis and Huxham; the adynamic and ataxic fevers of Pinel; the entro-mesenteric of Petit and Serres; the dothin-enteritis of Brettonneau; the Gastro-enteritis of Broussais, the Typhoid affection of Louis, Chomel, and Andral; Follicular Enteritis of Cruveilhier and Forgêt; the Typhoid entro-mesenteric of Bouillaud; the Enterite septicémique of Piorry.

It would be difficult to adduce a more signal instance of the transitory character of individual popularity than that which has struck us in the perusal of this excellent article in the work before us, written and published in Paris, where the facts and views which it records have been collected. It does not contain a single mention of the name of Broussais except as having given one of the synonyms prefixed to the chapter. Broussais, who but a very few years ago was in the mouth of almost every medical man in Paris, from the professor to the student, applauded by many, and combated or condemned by a few: Broussais, who told the crowds of his admiring pupils, that they might disregard all preceding authors from the time of Hippocrates to the present day, and throw them aside as mere ontologists, whilst the only necessary key to medical theory and practice was to be found in the physiological doctrine which he propounded to them—Broussais is not even once quoted. We looked in vain for the name of Broussais in the bibliography of the disease, in the exposition of its symptoms, and in the general and special instructions which are given for its treatment. Not a word of his exaggerated dread of aperient medicine—of his toleration of a loaded intestinal canal, that the fæces might serve as poultices to the ulcerated mucous membrane—of the myriads of leeches which he employed, or of the strict regimen which he prescribed. His merits and his defects are passed over in silence, and we are the more forcibly reminded of him by the very fact that so great and recent an idol is so completely thrown aside.

*Nature of the Malady.*

Typhoid Fever is anatomically characterised by lesion of an inflammatory nature situated in the follicular glands of the intestines and mesenteric ganglion. Louis regards this lesion as constant, whilst, according to Chomel, Andral, and Dalmas, it may in some cases be absent. Chomel having seen subjects sink when only one or two patches or only a part of a single patch of aggregate glands was diseased, was led to believe the possibility of the total absence of any lesion of the kind, and he was confirmed in this opinion by facts collected by Louis and Andral, in regard to individuals who had died after having presented many of the symptoms appertaining to Typhoid Fever, without any of the intestinal lesions which characterise it having been discovered on inspection.

"I stated," observes Dr. Grisolle, "in the first edition of this work, that I have myself seen two similar cases, but made the objection that the phenomena noticed were not precisely those which occur in Typhoid fever, and that they ought in consequence to be regarded as belonging to another disease, to an affection not as yet defined. I am still of the same opinion. I find amongst my notes a case, which on other grounds is important to the solution of the question before us; it is that of a man 22 years of age, who in 1835 died in the Hôtel Dieu, in the ward of M. Caillard, on the 27th day of continued fever, who had exhibited all the symptoms of severe Typhoid Fever, viz. intense headache (unaccompanied by epistaxis), vertigo, prostration of strength, absence of sleep, rambling, deafness, delirium, dryness of tongue, sordes of the mouth, diarrhœa, tympanitic distention of the abdomen, sibillant rattle, numerous sudamina (miliary eruption,) rose-coloured spots, gangrene of the sacrum, penis, and scrotum. On dissection, however, no characteristic lesion, either of the intestinal follicles or mesenteric ganglia was detected, but the spleen was enlarged and of a difluent consistence. This single fact would lead me to believe with Chomel that the intestinal affection is not an indispensable characteristic of Typhus Fever, since, in some excessively rare cases, it may be absent. Nevertheless, if the follicular affection of the intestines is not constant in the rigorous sense of the word, we repeat, again employing the expression of M. Chomel, that it is extremely rare for it to be altogether absent, and that there is not a single authentic case on record in which this lesion has existed, unattended by the symptoms of typhoid fever. One circumstance which has greatly contributed to excite doubts as to the importance of the derangement of the Peyerian glands is the assertion of medical men in London, Edinburgh, and Dublin, who aver that derangement of the intestinal glands is frequently absent amongst their patients, who, during life, had exhibited symptoms of typhoid fever. But it is now proved by the clinical cases collected in London by our friend Dr. Shattuck of Boston, and analysed by M. Valleix, as well as by the labours of Drs. Gerhard and Penwick of Philadelphia, that there exist in the United States and in England two febrile disorders, hitherto confounded together under the name of Typhus fever, but which are really distinct, and only resemble each other in their general symptoms. The one affects young subjects, and is the Typhoid Fever, such as we see it here (in Paris), the other common to all ages, with the exception of infancy, is Typhus fever. It is a disease distinct from typhoid fever; it is the typhus fever which we shall describe by-and-bye.

" It is a question whether the lesion of the intestinal follicles is a primitive affection, as in the case of most inflammations, or consecutive to a general state, like the eruption of small-pox, to which it has been compared. The first supposition has the greatest appearance of probability, when it is remembered that in most cases the abdominal symptoms, diarrhœa and colic, commence with the disease, which is not generally the case in other febrile affections. We cannot, however, speak with certainty as to the point.

" Does the intestinal affection constitute the sum of the disease? This does not seem to be probable, when it is known that there is frequently no proportion between the severity of the symptoms and the extent of the intestinal lesion. We have seen with M. Chomel death occur, although there was but a single patch of glands diseased, while, on the other hand, we often see in persons who have died from accidental causes very extensive lesions, although the symptoms during life had been of moderate severity. There are moreover, in the course of the disease, a number of morbid phenomena which are only explicable on the supposition of the existence of a general cause as yet unknown as to its nature and seat, which is placed by some in the nervous system, but which is more generally supposed to consist in an alteration in the blood, which has not yet been made out. Typhoid fever has sometimes been compared to eruptive fevers, and at others to simple inflammations, but if it have some points of resemblance with these, it has also many of dissimilitude. It is therefore expedient to consider typhoid fever a special disease, separated from all other affections by several fundamental characteristics."

The author then gives both sides of the question in the words of Professor Louis, who sums up the parallel, and the contrast between the eruptive fevers and the typhoid affection, and arrives at the conclusion adopted by our author.

Dr. Grisolle is one of those who admit the contagious nature of typhoid fever, although this character is disputed by many of his countrymen: but he agrees with Louis and others that it may be brought into existence by other causes, independently of contagion; and with Gendrin, that those who have been once affected acquire thereby a protection against future attacks.

Of the treatment he speaks under different heads. Under that of the Antiphlogistic he especially treats of the abstraction of blood. He admits its utility in certain cases, especially where there is a marked inflammatory character, but he condemns its general and copious employment, as adopted by M. Forget, and such as we remember to have been in vogue in the North of this island. He equally condemns the often reiterated bleedings of Professor Bouillaud. He gives the preference to leeches applied to the iliac region, if there be acute pain there, but he objects to their application to the anus, which he regards as not merely useless but liable to become a source of fresh irritation or even of gangrene. He likewise recommends the occasional application of leeches to the head, behind the mastoid process, but gives a salutary recommendation against allowing the bites to bleed long in children. He recommends the abundant use of drinks containing gum, or barley, as well as those which are flavoured with sugar and juicy fruits, some of which, we think, it would be better to omit, or give with great moderation. He enumerates emollient glysters, fomentations and cataplasms to the abdomen, and tepid baths, from which last, he observes, that great benefit is derived when the fever is violent, and the skin very hot and dry. He might have insisted more strongly on the efficacy of this treatment, and pointed out the circumstances which should direct its application, had he been acquainted with the classical work of Dr. Currie of Liverpool, and with that of Dr. Jackson.

He notices the contra-stimulant treatment merely to observe that he is unable to judge of the mode of employing tartar-emetic as recommended by Rasin, and that the use of sulphate of quinine in large doses and as a contra-stimulant is of questionable utility, although quinine, he admits, is decidedly useful in those cases in which regular paroxysms are established, when much smaller doses are employed.

Under the head of antiputrid or antiseptic treatment, he notices the employment of bark, camphor, musk, aromatics, wine, alcohol, mineral acids, internally and externally. He says of this treatment, that it was generally followed in France, whilst the opinions of Pinel (in the Nosographie Philosophique) were in vogue—that it has still many proselytes in England and Italy, and especially in Germany, but that it deserves no sort of confidence —that Andral has shewn that of 40 patients so treated 26 died, and that three only of the 14 who recovered appear to have been benefited by the treatment, and that the others recovered as they would have done if left to themselves. Nevertheless, he sanctions the use of wine in extreme cases, though on the more reasonable ground of combating prostration of strength rather than the tendency to putridity.

In treating of the evacuant plan the author observes, that medical men have alternately adopted and rejected the employment of evacuants in the treatment of continued fevers, according to the theory which they adopted respecting the nature of the disease. They were at one time generally abandoned, and Brettonneau and Lerminier, who were almost alone in adhering to the older doctrines, were unable to inspire their brethren with the confidence that purgatives might be brought into contact with ulcerated intestines without danger. More recently, M. Delarroque, physician to the Hospital Necker, has demonstrated how groundless were these fears, and proved, by a numerous series of well-observed facts, the advantages of evacuants in the treatment of severe fevers. This physician administers aperients in all the forms and periods of the disorder through its whole course up to complete convalescence. He generally commences by an *emetico-cathartic*, which Dr. Grisolle does not consider as useful, except there be symptoms of gastric oppression. The patients take every day a bottle of sedleitz water, or thirty grammes of castor oil, or two grammes of calomel, or a dose of cream of tartar. Pains in the belly, cholic, diarrhœa, and materorismas are, in his opinion, far from contra-indicating the use of purgatives, but seem, on the contrary, to call for their employment. If, in extraordinary cases, they increase the pain or produce hyper-catharsis, they are to be suspended for twenty-four hours. Delarroque conjoins with these means sweetened drinks and poultices on the belly, and gives tonics as soon as the fever is turned. In adopting this treatment, Delaroque has only lost one patient in ten. This plan has been tried by Honoré, Gueneau de Mussy, Bricheteau, Beau, Piédagnel, Jadioux, Andral, Louis, and many other physicians in Paris, who have all acknowledged the good effects of the evacuant treatment. Louis, after analyzing the different methods of treatment adopted in typhoid fever, gives the first place to the evacuant plan. It not only diminishes mortality, but shortens the duration of the disease. Grisolle adds, that by employing purgatives we are not favouring the development of any complication, and that the two most dreaded accidents, hæmorrhage and intestinal perforation, are much more rare than when the other modes of treatment are employed. He states that, in France, castor oil or sedleitz water is generally prescribed. In Germany and some parts of Switzerland calomel is most in favour, and this medicine is also preferred by Lombard and Fauconnet, who declare that they have only lost nine patients in a hundred so treated. He notices the assertion of Barthez, Rilliet and others, that the purgative plan is inapplicable to infants, though useful in adults ; but he regards the assumption as doubtful, and worthy of further enquiry.

It is remarkable that the author makes no allusion to the employment of purgatives in this country, where, since the publication of the important work of Dr. Hamilton, they have been so generally employed, that the prescriptions, and books, and records, of almost every medical man in the country during the last thirty years might be appealed to in proof of the general adoption of the very plan to which the author has given such decided preference. In fact, the use of purgatives has not unfrequently been a subject of reproach against us on the part of our continental brethren, and it has been one of the happy results of the eminently eclectic character of British medicine, and of our readiness to profit by the observation and experience of our contemporaries, that the immoderate employment of purgatives has been checked, without our falling into the opposite error of rejecting them.

*Typhus.*—It has been attempted to constitute a disease distinct from typhoid fever under the name of "Typhus, or Plague of Europe." The fever so designated has been seen in prisons, hospitals, and camps, and has been common in besieged cities, wherever numerous individuals are brought together in close situations or under other unfavourable circumstances. It is eminently contagious, which has been regarded as one of the grounds of distinction by those who doubt the contagiousness of the typhoid affection. Many of the symptoms are admitted to resemble those of the typhoid affection. It has been attempted to shew that the derangement of the alimentary canal is slight or altogether absent in many cases of typhus. That petechiæ are much more common in typhus and miliary eruption than in typhoid fever—that the red lenticular spots appear some days earlier in the former—that hæmorrhage and intestinal perforation are also more uncommon, and that the spleen is not essentially in a morbid condition. Notwithstanding these attempts at distinction, the author regards it as proved by the pathological researches of Chomel, of M. Gaultier de Claubry, and by the narratives furnished by M. Landouzy himself (who adopts the opinion that they are different), that these two affections are essentially the same, and that the supposed differences merely belong to the accidental varieties of different epidemics.

The case is different with the typhus or continued fever in England and Ireland. The medical men of Great Britain, and even French physicians who have had the opportunity of visiting British hospitals, have noticed cases of fever in which the lesion of the Peyerian gland was absent. At first the French physicians of the school of Louis were disposed to doubt the accuracy of such narratives, and to presume that the inspections had not been made with sufficient care. But the concurrent testimony of several able and experienced observers, and more especially of Drs. Gerhard and Shattock, American physicians, who had studied in Paris, removed all doubt on the point, and have led to the conclusion adopted by Professor Louis, as well as by the present author and many others, that there is a really distinct malady not uncommon in Britain, and occasionally occurring as an epidemic in the United States, which, notwithstanding many symptoms common to it and the typhoid, it is essential to distinguish from that form of fever. Whilst the most important anatomical difference is observed in the absence of lesion of the aggregate glands, a difference is also to be noticed in the cutaneous eruption occurring in the two diseases. In typhus fever the eruption consists in numerous, irregularly grouped spots, varying in size from that of the head of a pin to that of a pea, of a violet or deep red colour, and not disappearing under pressure. It appears on the sixth or the eighth day, and rarely disappears before the twentieth, but sometimes persisting to the twenty-eighth, or even thirty-first day. Miliary eruption is said to be uncommon in the English typhus fever. Blood drawn from the veins has no buff, and the clot is soft and again becomes fluid. Epistaxis is much less common than in typhoid affection.

If, with Dr. Grisolle, we have made the distinction between the typhoid affection as characterized by the specific derangement of the aggregate glands and the cases of fever noticed in England and America, which we think must be conceded, various considerations not altogether unimportant necessarily suggest themselves. If, in these days of careful anatomical research, the distinction has been with difficulty established, how are we to receive the narratives of the best older authors who have written on fevers, and who, with the same varieties of disease under observation and treatment, were incompetent to draw the distinction? If the derangement of the aggregate glands characterizes a disease as distinct and specific as scarlet fever or measles, and which is wont in its progress to be accompanied with many symptoms in common with typhus fever, are we prepared to admit that the cases in which the derangement of these glands has been absent, as in the fevers noticed in Britain and the United States, as really constituting a second specific form of fever, and are there not, rather, sufficient reasons for concluding that the conditions and symptoms, regarded as common to typhus and typhoid fever, may present themselves in several febrile diseases in common, constituting, as the older authors led us to believe, the tokens of a particular stage in the morbid process? Such is the idea which we are disposed to adopt, and if it will not remove the whole difficulty, it will at least prepare us to appreciate the importance of those accurate pathological researches which alone can enable us to distinguish different forms of fever tending to some common results.

The other forms of continued fever which are noticed are the bilious fevers of warm climates—yellow fever and plague, which we pass over. The eruptive fevers constitute the next group. In speaking of variola, he notices Piorry's plan of softening the skin by baths and moist compresses, and then breaking the pustules to prevent the formation of scars; and also the plan of cauterization advocated by Brettonneau, Seres and Velpeau; but these plans are not commended by the author. He has a better opinion of the application of mercurial ointment.

In speaking of Vaccinia, he admits the deterioration of the vaccine virus since its introduction by Jenner, and recommends frequent recurrence to the original matter from the cow, which produces a much finer pustule, attended with more constitutional affection than is the case with the older virus. He says that, by inoculating the cow with old virus from the human subject, we do not obtain any advantage; but he says nothing of the means of re-producing efficient vaccine virus, as taught by the remarkable researches of our countryman Mr. Ceely, or rather he merely alludes to them to express a doubt of their accuracy.

In treating of modified Small-pox and Chicken-pox, he alludes to the opinion held by the late Dr. Thomson and Mons. Rayèr, that modified small-pox and chicken-pox are only varieties of the same affection, being both produced by the contagion of small-pox. He does not consider the question as fully decided, though he inclines to the opinion of their being distinct affections. The fact of chicken-pox being seen in those who have had neither small-pox nor cow-pox concurs with the characteristic appearance of the eruption to place chicken-pox as a disease wholly distinct from small-pox. In his article on Measles he does not agree with Willan, that this disease was known to the Greeks and Romans, but thinks that it was probably derived from Asia, the apparent source of so many of our diseases, at a period not known, since Rhazes, the first author who has given an exact description of it, does not describe it as a new disease. He notes as varieties—measles without catarrh—and measles without eruption,—and the black measles of our countryman Dr. Willan, to whom he frequently refers in the course of his work. He mentions the formidable complications of measles; namely, Pneumonia, which in infants is almost always of the lobular form—Enterocolitis—Gangrene of the lips and lungs—Inflammation of the membranes of the brain, with Delirium, Coma and Convulsions, and Hooping Cough and Croup, which often succeed to it. He does not omit to mention the curious fact pointed out by Hunter, that the contagion of small-pox and measles may simultaneously exist in the patient at the same time, and produce their characteristic eruptions, but that measles is suspended whilst small-pox proceeds with its development, and then resumes its ordinary course. The measles greatly favour the production of consecutive tubercles. And, measles attacking those already affected with phthisis, the progress of this disease is accelerated. Cold, he thinks, produces in those who are convalescent from measles, anasarca much more frequently than after scarlatina.

Measles, on the other hand, have sometimes been seen to produce a favourable effect on pre-existing maladies. Thus Rayer gives an example of chronic Exema of the face, and Alibert one of Empetiginous exema of the scalp, which were quickly cured after the recurrence of measles. He notices the inoculation for the disease by means of blood taken from the spots, as practised by Home in Edinburgh, and Speranza at Milan, which caused the appearance of the disease after six days of inoculation, and also inoculation with tears or saliva, which does not appear to be quite so effectual.

We pass over the other eruptive fevers, to notice the important division of Intermittent Fevers, which he distinguishes as simple, mild, or pernicious, manifest or masked, essential or symptomatic, regular or irreglar.

### Simple Intermittent Fever,

Having no local seat, furnishes no pathological appearance after death which can be regarded as the starting-point for the phenomena noticed during life. The enlargement of the spleen, which is almost never absent, being rather to be regarded as a concomitant affection. The softening of this organ is only to be met with in those intermittents which are pernicious.

The types of intermittent fever, distinguished by the returns of the paroxysms, are the quotidian, tertian and quartan, with their varieties, double quotidian, double tertian, and double quartan, but these last, with the exception of the double tertian, may be regarded as either exceptional or altogether doubtful; and the same may be said of triple tertian, triple quartan, and also of the quintan, septan, octan, monthly and annual, which have been admitted by some nosologists.

It is needless to quote descriptions of the different stages, which, though good and concise, offer nothing remarkable in relation to facts which are sufficiently known. A comparison of the frequency of different types is interesting. From a comparison of more than 160,000 cases observed in different countries, it appears that quotidians are more common than tertians, in the proportion of 9 to 1, and that quartans are so rare that there are scarcely two or three in a thousand. The author does not agree with Boudon that there is any connection between the type of the fever and the intensity with which the malaria is disengaged.

The preparations of bark hold, of course, the first place among the curative means, and the sulphate of quinine is preferred to all the others. Besides the ordinary mode of giving it by the mouth, Dr. Grisolle notices its administration as an enema, which is less certain than the endermic employment, as tried by Chomel. The sulphate of quinine applied to the skin only two hours before an expected paroxysm, succeeded in setting it aside, but a much longer time was required when the remedy was given by mouth. The application to the skin had the serious inconvenience of producing sloughs and ulcers, both painful and slow of cure. He notices, besides quinine, a list of other articles, such as salicine, chesnut bark, opium, tonquin bean, orange-peel, digitalis, armica, iron, mercury, alum, salt, phosphorus, all of which he rejects as entitled to no confidence. He has some reliance on arsenic, but he appears to regard it as so dangerous as to be only admissible in the smallest doses in those cases which resist the influence of quinine.

Our cautious author rejects the attempts to localise the affection by placing it either in the intestinal canal where cadaveric changes have been mistaken, or in the spleen, where Piorry and Audouard would fix its seat.

"It would be idle," he says, "to enter into a discussion to prove that intermittent fever is not an inflammation. Shall we, with Giannini, call it a *nevrodsthenie,* or, with Brachet and Rayer, a *neurosis,* a cerebro-spinal irritation, with Maillot, or with Worms, an affection of the ganglionic system? It seems reasonable to connect the principal symptoms of the disorder with nervous derangement. We are not, however, in possession of any positive knowledge on this subject, and it is much better to confess our ignorance than to attempt to conceal it under ill-defined pretensions. It has not merely been attempted to localise the derangement in intermittent fever, but also give an explanation of its periodicity. The opinions which have been advanced in relation to this question are so extravagant that we think it best wholly to pass them by.

"In fine, we must admit that we know nothing of the composition of miasmata, of the organ upon which they act, or of the manner in which quinine operates to neutralize their influence."

### Pernicious Intermittent Fevers.

The term pernicious is applied to those intermittent fevers which, on account of their great severity, and rapid progress, may terminate fatally in a few paroxysms. There are several varieties of pernicious fevers. In some there are many severe symptoms without the predominance of any one. The countenance is remarkably changed—the strength is prostrated —the pulse is small and irregular—the tongue dry, and the intellectual faculties blunted. More often, there is some symptom which arrests particular attention. Sometimes the cold stage is so intense that the countenance appears cadaverous;—the shivering is extreme, the breast cold, the voice gone, the thirst great, the pulse small and irregular. Death may take place in the first paroxysm, but if this be survived the warmth is slowly and imperfectly restored, and without prompt assistance the second attack is generally fatal. In the next variety death occurs in the sweating stage. This is a more insidious form, the case at first appearing mild, but in successive paroxysms the sweating becomes increasingly profuse, and soaks every part of the bed; the patients become cold, their strength is exhausted, their pulse extremely small, but the intellect is not affected.

### *Class* 2.

In the second class, or that which comprises those diseases which consist in a disturbance of the due proportion of blood, we have first the cases of excess of blood constituting plethora and local congestions, which are either active or passive. He properly distinguishes this state from that of inflammation, though he regards it as one of the modes in which inflammation may be set up.

The opposite state furnishes the character of the second division of this class, in which are placed Anæmia, Chlorosis, and the Anæmia of particular organs. With respect to general Anæmia, he refers to the researches of Andral and Gavarret, Bouillaud, Marshall Hall, and Piorry. He mentions the views of Drs. Hope and Ward, respecting the peculiar sound termed *bruit de diable,* which is heard in patients labouring under anæmia, but he does not agree with them in the opinion that it is produced in the larger veins, having been shaken from this opinion, which he had once adopted, by the observations of Mons. Beau, who maintains the old opinion that the sound is arterial.

Anæmia not being solely the consequence of loss of blood, but originating in many instances in serious visceral disease, the treatment must of course be various, as well as the probability of success. He gives the first place to ferruginous preparations, as not only contributing to improve the blood, but also as the most effectual remedy to the neuralgic pains which are apt to occur in the course of the disease.

Chlorosis is regarded as essentially a state of anæmia, and not necessarily distinguished as a special affection, yet the class of subjects to whom the affection is peculiar seems to render the division not altogether unsuitable.

The cases of partial anæmia are merely alluded to, the author observing "that, in the present state of our science, we know absolutely nothing about them."

*Class* 3.

The third class, or that of Inflammations, in which are included not only inflammations of the viscera, but also various forms of skin-disease, occupies a considerable part of the first volume.

The several special affections are arranged in the following groups:—

1. Inflammations of the digestive organs.
2. Inflammations of organs accessory to digestion.
3. Inflammations of the organs of respiration.
4. Inflammations of the organs of circulation, including angioleucitis, or inflammation of the lymphatic vessels.
5. Inflammations of the nervous system.
6. Inflammations of the organs of the senses.
7. Inflammations of the urinary organs.
8. Inflammations of the genital organs.
9. Inflammations of the cellular membrane.
10. Inflammations of the skin.

*Class* 4.

In speaking of Hæmorrhages he distinguishes the spontaneous from the tromatic, which fall to the care of the surgeon. The spontaneous are either symptomatic or essential; the former being referrible to some anterior affection, of which it may be considered as forming a part, but, in the latter, the hæmorrhage itself constitutes the disease. These hæmorrhages are distinguished by authors as active, passive, constitutional, accidental, succedaneous, critical, internal, and external. They are also either interstitial or on the free surfaces.

There is not always any manifest erosion by which the blood has escaped from its vessels, and in such cases it seems necessary to admit an alteration both in the fluids and solids, but it is impossible to state the intimate cause of such a phenomenon. When death has been caused by profuse hæmorrhages, the sanguiferous system is found almost empty—the blood which it contains is pale and serous—all the organs are reduced in colour, and the heart is contracted on itself. M. Beau has lately advanced an opposite assertion (*see Archives de Medicine*, 1845), viz. that, in those who die after having suffered from repeated hæmorrhages, the heart is hypertrophied and dilated. The fact is far from being established on positive proofs, and at present it has scarcely any support except from a few experiments performed on inferior animals.

We may pass over most of the forms of hæmorrhage, which are arranged very much in the order adopted for the corresponding inflammations. A single page is allotted to hæmatidrosis or bloody sweat, of which it is observed, "there exist a certain number of well-authenticated observations to prove that blood has sometimes been exhaled from the skin, probably by the same course as the perspiration. Such hæmorrhages are rarely general—they are partial, and are seen on those parts on which the skin is white, fine, and often bathed with sweat"—"or they may take place on the surface of an old cicatrix."

The cases alluded to are such as having occurred in young and middle-aged women, were connected with amenorrhœa or deficient menstruation; but no mention is made of another form of hæmatidrosis, which has in some rare cases occurred in conjunction with great disturbance of the circulation, arising from moral or morbid causes affecting the heart. A careful commentary on such instances is to be found in the recent and remarkable work of a learned and accurate English physician, Dr. Stroud, who, in treating on the Physical Cause of the Death of Christ, has had his attention directed to this symptom related by Luke, as accompanying the agony of our Lord in the Garden of Gethsemine.

Of the interstitial hæmorrhages to which the name of apoplexies has been given, cerebral apoplexy is the most important. After noticing the well-known fact that, the paralysis consequent on cerebral apoplexy occurs on the side of the body opposite to that on which the effusion of blood on the brain has taken place, the author says. "There exist, however, some authentic observations of paralysis taking place on the same side as the effusion, but these cases are too decidedly exceptional to prevent our stating, in a case of hemiplegia, that the seat of the effusion is in the opposite hemisphere. Is it possible to localize the malady more precisely? Can we for example, assert that the effusion is in the middle or the anterior lobe, in the corpus striatum or the thalamus opticus, or in the cornu ammonis, or in the convolutions from indications afforded by the seat and limits of the paralysis. I regard this localization as impossible in the present state of Science." Though he founds his opinion on the numerous observations of Andral and Finck, who arrived at this conclusion, we must admit that we still attach great confidence and importance to the views of Dr. Foville, who, though not named, was with his friend Delaye, the author of the localization in question. Our own experience has shewn its accuracy in many instances. Dr. Bright and the late Dr. Sims have furnished examples which tend to confirm it, and lastly the very cases which are adduced by its opponents, when sufficiently detailed, offer the explanation of the apparent exception. As a general remark, it may be observed, that this localization cannot be made or relied upon in many recent cases, since the parts in the vicinity of the clot may suffer to a degree which interferes with the function, although the absolute lesion may not extend to them. In the process of recovery there is a marked difference in the returning power of the paralyzed members corresponding with these uninjured portions of the brain.

The *fifth class*, or that comprising the morbid secretions, relates first, to the serous effusions of Dropsies. The introduction of Asiatic Cholera into this division appears to be of questionable propriety. Secondly, to the mucous secretion of which bronchorrhœa, gastrorrhœa, catarrhal diarrhœa, leucorrhœa, and vesical catarrh are particularized. Thirdly, the proper secretions of special organs, such as the saliva, the bile, the urine, milk and semen; and fifthly, morbid secretions, all the examples of which group consist in the preternatural development or accumulation of air or gas.

In the *sixth class*, which comprises the effects of poisons, the author adopts the classification of Vicat, modified by Orfila, and makes four divisions according to their mode of action upon the system: viz.—1, Irritating Poisons; 2, Narcotics; 3, Narcotico-Acrid; and 4, the Septic or Putrescent. Though the most convenient classification, it has its defects and difficulties; and the author refers to the remark of Mead, that the same poison will act differently according to the dose and mode of application, and cites in illustration preparations of lead, which, though ranked with irritating poisons, exert a widely different influence after their absorption. "Many poisons are taken into the alimentary canal; others act when they have been applied to other mucous membranes, or to the skin, more especially when denuded; or they may have been applied to a wound or introduced into the cellular membrane."

Whatever may be the mode of introduction into the economy, poisons may be limited to a local action, irritating or disorganising the tissues—such are the concentrated acids, potash, &c. Others producing no effect on the surfaces with which they are brought into contact, act on more or less distant organs after they have been absorbed. This is particularly the case with opium. Some poisons have a complex action. They first inflame the structure to which they are applied, and then, a portion being absorbed, occasion similar lesions in other organs. Such, for example, are cantharides, which, being swallowed, inflame some parts of the urinary passages. Others exert their force on the nervous system, either causing excitement or insensibility, without producing any material change of tissue. Such is the action of nux vomica and tobacco. Lastly, there are some poisons which, being absorbed, act primitively upon the blood and change its composition. Many poisons, but especially the several kinds of morbid virus, are of this description.

We see, then, that a great number of poisons are absorbed. This absorption, which but a few years since was unknown in the case of some substances, and rather admitted on physiological inference in the case of others, has been demonstrated by experiment by Orfila, who has discovered most mineral poisons in the tissues of several organs, in the blood, and in the urine. A new path has thus been opened for legal medicine.

Most poisons act immediately, but others produce their specific effects after the lapse of days, weeks, or months, requiring what is termed a period of incubation, as in the case of poisoning by different kinds of morbid virus. The symptoms which are induced by poisons have generally an acute or hyper-acute character, and some persons have even denied the existence of slow or chronic poisoning, yet we have undoubted instances of this kind in the influences of lead or mercury. The old notions of a slow poison by which death may be insured at a fixed distant period, was an idea produced by ignorance and maintained by popular prejudice, and may be regarded as contrary to the laws of nature. Chronic visceral disease, either produced spontaneously or the sequela of an acute affection induced by poison, may have been frequently mistaken for cases of slow poisoning.

Symptoms dependent on disease of the abdominal viscera and derangements of the cerebro-spinal system may bear some resemblance to the effects of poison, as in indigestion, iliac passion, cholera, peritonitis, hepatic and nephritic cholics, certain nervous affections, and apoplexy of the brain and spinal cord. In general, inspection will remove the doubts which may exist; but when the investigation of the symptoms, and the inspection, aided by the microscope, are insufficient, recourse should be had to a chemical analysis of the excretions, and likewise of the organs themselves.

We pass over the author's remarks on individual poisons of the acrid narcotic, and narcotico-acrid classes, and give his enumeration of the putrid or septic poisons. We may, however, just observe that, in treating of poisoning by arsenious acid, he confirms the recommendation of the hydrated oxyde of iron. If this be not at hand, the *aperient saffron of Mars* may be substituted for it, but in larger doses. The antidote is to be given after the mechanical irritation of the fauces has been employed to produce the rejection of the poison by vomiting. We have employed the hydrated oxyde of iron in a case in which about two drachms of white oxyde of arsenic had been taken. Success in this instance was complete, the patient being perfectly well in the course of a few hours. But in this case repeated powerful evacuation of the stomach had previously been produced, by forcing the patient to swallow several raw eggs, then dosing him with a solution of the sulphate of zinc, followed at intervals by copious draughts of warm water. The energetic and copious vomiting brought away considerable quantities of arsenic in substance. We have known the white oxyde of arsenic to be retained, after an emetic of sulphate of zinc had been employed in the usual mode; which is not surprising, when it is considered that one of the effects of the arsenic, taken in substance, is to excite the effusion of lymph, by which the arsenic is entangled, and retained in contact with the stomach. This will account for one of the most remarkable cases on record, in which a young woman survived the swallowing and retention of a large dose of white arsenic, which, some months after, was discovered shut up in a membranous cyst attached to the lining of the stomach.

The effects of alcohol are mentioned among those of vegetable poisons, and the general or partial combustibility of the body is recognised as one of its rare but undoubted effects. When general, this combustion may be rapid, and completed in two or three hours, or even in a single hour. This form is mostly met with in aged females, whilst the partial combustion has been more often noticed in men. The simple effusion of cold water seems to accelerate, rather than stop, the combustion, which, however, may be subdued by prolonged immersion in very cold water.

Under the head of Septic Poisons, the following are included :—Putrid exhalations; putrid articles of diet; putrid matters applied to wounds; ergot; maize; gas from cess-pools and drains; animal poisons, as of the viper, the rattle-snake, the scorpion, the wasp, &c.; the several kinds of morbid virus, as of syphilis, yaws, hydrophobia, malignant pustule, and farcy or glanders.

### *7th Class.*—Lesions of Nutrition.

The subdivisions under which the author arranges the affections comprised in this class are,

1st. Hypertrophy, specifying that of the brain, spinal cord, heart, liver, spleen, thymus and thyroid glands, and Arabian elephantiasis. Cirrhosis is introduced as hypertrophy of the liver, but constituting a separate form.

2ndly. Atrophy—viz. of the brain, spinal cord, heart, liver, and biliary ducts.

3rdly. Induration—of the brain, spinal cord, heart, and liver.

4thly. Softening—of the brain, spinal cord, heart, stomach, intestinal mucous membrane—of the liver, spleen, uterus, and bones.

5thly. Gangrene—specially noticing that of the lungs, the mouth, pharynx and vulva.

6thly. Ulceration.

7thly. Those lesions which produce the contraction, obliteration, dilatation, perforation and rupture of hollow organs. Under this head are noticed the openings of the heart, contraction and obliteration of arteries, veins, and sinuses; contraction of the air-passages of the œsophagus and intestinal canal; dilatations of the bronchi and pulmonary cells; dilatations of the heart and of the lymphatics, aneurisms, dilatations of the pharynx, œsophagus and stomach—Ruptures of the œsophagus, stomach, intestine, and parietes of the heart; Ruptures of the tendinous cords of the heart; Preternatural communications between the cavities of the heart; Ruptures of the aorta; rupture of the lungs and pleura, producing pneumo-thorax; rupture of the diaphragm; rupture of the spleen, liver, kidneys, and gall-bladder; ruptures of the urinary bladder and of the uterus.

### *Class* 8.

In this class, which comprises structural changes and accidental productions, there are two principal divisions—the first consisting of those diseases which are characterized by the transformation of tissues, or by the development of new tissues analogous to or identical with those which are natural to the body;—the second are marked by the existence of new products, altogether abnormal.

The first of these divisions comprises fatty, serous, corneous, cartilaginous, and osseous formations, and some forms of polypi. In the second class are included inorganic concretions, entozoa, cancer, tubercle, and melanosis.

In speaking of cysts, which he regards as the accidental production of a serous tissue, he notices their occurring sometimes solitary and sometimes in great numbers.

"We sometimes," he observes, "find so many in the same individual, that it has been supposed that their production depends on a particular diathesis. Such is the case of a woman related by Morgagni, in whom he found 800 serous cysts, disseminated in most of the viscera.

"The mode of formation of cysts has been a subject of much discussion. Some consider, with Louis, Haller, and Morgagni, that the effused material is pre-existent to the cyst, whilst, according to Bichat and Cruveilhier, the opposite takes place. The latter opinion is probably more generally correct. It is, however, indisputable, that in many cases the formation of a cyst is consecutive to the presence of a foreign body. Such, for example, we have seen to be the case with collections of pus and blood in the brain. It is also seen when bullets are lodged in the substance of tissues."

Cruveilhier's arrangement of cysts, which is very good and practical, recognizes these and various other forms of cysts.

In speaking of particular forms of cysts he notices specially those of the brain, those of the liver and spleen, those of the kidney, the dilatation of the pelvis of the kidney, caused by some obstruction in the passage of the urine to the bladder, to which the name of Hydronephrosis has been given by Rayer, who has well described the affection, Ovarian Cysts, which he divides into the hairy, the serous, and the acephalocystic.

Ovarian cysts, containing hair, are often accompanied by teeth, generally those of the first dentition, portions of bone, apparently belonging to different parts of the skeleton, skin, muscular tissue, and a greyish-white matter.

"The origin of these productions has been much disputed, but we think, with Haller, Cruveilhier, and Velpeau, that they appertain to fœtal debris. It must be admitted that, in the cases of which we are speaking, ovarian pregnancy had taken place; that the fœtus, having perished at a more or less early period from conception, had been almost entirely dissolved, while certain parts had resisted destruction, or even continued to grow. Such are the hair and even the teeth, which sometimes acquire considerable development. The interest of these tumours is almost wholly of an anatomico-pathological character, as they generally remain stationary during life, producing little distention or derangement of health. They may however be accompanied by inflammation, and attended with symptoms observed in other cases of extra-uterine pregnancy."

We do not admit the author's explanation of the origin of these cysts as applicable to all cases.

Of the serous form of ovarian cysts he speaks at greater length.

"Serous cysts, which are rarely met with in other parts of the body, are very frequent in the ovaries. The peculiarity is explained by the structure of these organs, which contain within their interior fifteen or twenty small vesicles, which are probably the most frequent starting-points of the malady. The tumour, however, does not always begin exactly within the ovary. Sometimes it is external to it; that is to say, immediately under the peritoneum, but without the fibrous coat. It is even not unfrequently situated upon the uterus, or some other of its appendages, besides the ovaries. However some persons believe that, even in these cases, the ovary is the original source of the disease. They suppose that an ovum spontaneously detached under the influence of venereal excitement has fallen into the peritoneum, where it has contracted attachment, and continued to grow.

" This opinion, of which the accuracy cannot be demonstrated, is nevertheless very probable, for cysts within the pelvis are perhaps never met with but in the female. It is therefore natural to refer the production of these tumours to some natural organic condition of the female, and this condition can be no other than the presence of an organ which does not exist in the male."

Without questioning the strong predisposition which exists in the ovary and its vicinity to the production of serous cysts, we cannot unite in the theory here adopted, since cysts having in every respect the anatomical character of those occurring in the ovary are found in various organs and textures of the body, in males as well as in females. That they are not degenerations of the vesicles of De Graaf, is evident from the fact, that the morbid enlargement of these vesicles, when it does occur, gives rise to cysts materially different from those which constitute the ordinary form of ovarian dropsy. It is not, however, improbable that the peculiar microscopic texture of the ovary, which admirably fits it for the production of a succession of Graeffian vesicles, may, by some disturbance of this process, bring about the formation of the cysts in question.

A good description is given of the symptoms, varieties, and terminations of these ovarian cysts. A case observed by Dr. Bonfils, in which the rupture of the cyst was followed by radical cure, is mentioned as a solitary instance. Several instances of the same kind, have, however, been seen and recorded in this country.

With regard to the treatment, our author admits the entire failure of all medical means. The strong support and compression of the abdomen is to be adopted as a palliative. Puncture, generally employed with this view only, has sometimes obtained a radical cure. The puncture, which is generally made through the abdominal parietes, may sometimes be performed through the vagina, which is not only favourable to the escape of the fluid, but also to the introduction of the canula, by which a gradual and continued discharge may be procured ; and such evacuation has been known to lead to an entire cure. The operation is only practicable in this mode in some cases, and when practicable is not always safe.

Some persons have ventured to attempt a radical cure by injecting irritating liquids or vapours into the sac after evacuation, and others have incised the cyst after having caused adhesion to the abdominal parietes. All of these plans our author rejects as more or less imprudent, and whatever success may have been related as accompanying them is not sufficient for their justification. On the same grounds he rejects the extirpation of the cyst after merely alluding to the operation, and stating that it has been performed. He, however, recommends a treatment stated to have been successfully performed by Professor August Bérard. It consists of making punctures through the cyst with a long acupuncture needle. He considers that, by this means, the cyst is so modified as to promote a slow but ultimate absorption of the liquid.

Amongst the horny and epidermic productions are noticed horns, which are more often met with in aged females than in males. They are sometimes solitary, more seldom numerous. They are about two centimetres in length, but have been seen upwards of thirty. Malpighi and Raspael thought they were formed by a nervous prolongation from the dermis, but Breschet thinks, with more probability, that they are a morbid secretion. The other forms of corneous production are ichthyosis, pityriasis, lepra, and psoriasis.

After noticing cartilaginous and osseous formations, Dr. Grisolle speaks of Polypi, merely enumerating those which from their position come more particularly under the attention of the surgeon, as for example, in the nose, pharynx, and uterus, but dwelling on those of the stomach and intestines, and of the larynx and bronchi. He states that most of the cases of polypi of the heart which have been published in France and England, are only cases of sanguineous concretion, but that there are some well-authenticated though rare examples of accidental productions of the same character, or analogous to the fibrous and fungous polypi of the nose and uterus. Their most frequent seat appears to be the left auricle, and in two instances they were attached to the spot at which the foramen ovale had been closed. In two cases, which we have seen, adventitious productions somewhat resembling this character were attached to the margin of this valve, which had remained open to an advanced period of life.

The so-called vegetations of the heart noticed by Corvisart and Laennec, and which the former, erroneously supposed to be of the character of syphilitic warts, are formed in the ventricles, or attached to the valves. Some are regarded as unquestionably the result of endocarditis, whilst others have more the character of coagula.

ACCIDENTAL PRODUCTIONS FOREIGN TO THE SYSTEM.

We shall pass by all the calculi and the parasitical animals which are included by the author in this class, only observing that he inclines to the recent and not improbable opinion, that acephalocysts are merely the envelopes to the echinococcus, and that the vesicular mole or placental hydatid is to our surprise included under the head of hydatids, which is the more remarkable as the author is acquainted with the researches of Madame Boivin.

In the same section, and without any subdivision, the author treats of the whole group of cancerous scrofulous and melanotic productions, these last being separated from the other forms of malignant disease. In noticing the microscopic characters of cancer, as marked by various forms of nucleated cells, he only mentions the observations of Lebert, without alluding to those of Müller, which were, we believe, the first, and which have been followed up by many others.

There is nothing new or remarkable in the description which is given of the various forms of cancer, and which appear to have been drawn from various sources, including several English authorities. The author adopts the opinion of Bérard with regard to the softening process, depending on increased vascularity, which is doubtless a part, though we believe not the whole of the truth. He agrees with those who of late have properly distinguished the colloid or gelatiniform cancer from the encephaloid. The distinction is doubtless correct with respect to the most characteristic specimens in which there is no blending of the two diseases, but there are examples in which, judging from external and manifest appearances, they seem to concur and insensibly pass into each other.

Cancer is described not only in its general characters, but in relation to the most important organs liable to be invaded by it, such as the lungs, pleura, stomach, intestines, liver, uterus, &c.

The author treats of tubercle in general as a peculiar deposit, following Laennec and Louis for its manifest characters, and Lebert for the microscopic. He notices the views of Baron, Cruveilhier, Lallemand, Piorry, and Carswell, as to the mode of origin, and those of Broussais, Lombard, Andral, and Louis, as to the mode of development and the influence produced on surrounding parts.

Under the special affections of this group, phthisis pulmonalis is of course the most prominent, and the article on this disease contains a summary of the observations of the best authors, but he does not appear to be aware of the beautiful microscopic observations and researches of our countryman, Mr. Raine.

Tubercular meningitis, which forms another member of this group, is made to include hydropcaphalis internus. Tubercles in the substance of the brain are treated of separately.

Tubercles in the mesenteric glands, constituting mesenteric atrophy, forms another division under the name of Carreau.

The article on scrofula is short, and we regret to observe the absence of any notice of the laborious enquiries of our countryman Benjamin Phillips.

We pass over the *9th Class* of nervous affections to notice the last, viz., *10th Class*, in which are placed affections peculiar to certain organs or tissues. This comprises the following list:—

Disorders peculiar to the digestive organs, the first of which are those accompanying the first dentition; secondly, Indigestion; thirdly, Gastric disturbance; fourthly, Intestinal disturbance; fifthly, Constipation; sixthly, Intussusception, or volvulus; seventhly, Diabetes.

Peculiar to the liver—Jaundice or Icterus.

Peculiar to the kidneys—Bright's disease.

Peculiar to the heart—Insufficiency of the valves, viz. of the Sigmoid, of the tricuspid and mitral.

Those peculiar to the air-passages;—Asyphyxia, divided into three forms—1, by compression of the chest,—2, by strangulation,—3, by submersion.

Disorders peculiar to the genital organs;—Dysmenorrhœa; Irregular menstruation; Amenorrhœa; Cessation of Menstruation, or symptoms attending the change of life.

Disorders peculiar to the fibrous and muscular tissue:—Rheumatism, subdivided by its several seats (one of the most remarkable of which is rheumatism of the uterus during pregnancy and parturition); Acute rheumatism and rheumatic gout; Chronic rheumatism.

Diseases peculiar to the skin:—Prurigo, lichen, urticaria, elephantiasis, lupus, maculæ, lentigo, eplelides.

Were we disposed to criticise the arrangement adopted by the author we should, perhaps, object, in the first instance, to the formation of this last and miscellaneous class; and secondly, to his having placed in it some affections which might have been arranged in some one of the preceding sections, to which they would seem naturally to belong. Little practical inconvenience can, however, result from the course which the author has adopted, and the practitioner, as well as the student, will find the articles on Bright's disease, on diabetes, and on rheumatism, very worthy of attention. In the short article on deficiency of the valves of the heart, he gives a prominent place to the labours of Corrigan, Hope, and Henderson, as well as to those of his countrymen, but he does not seem to be aware that the affections to which he alludes were known and described before the appearance of the articles to which he refers.

In taking leave of the work we think we are justified in pronouncing it one of the very best and most comprehensive general works on medicine with which we have yet met. The abundant proofs which the author has given of his labour and candour warrant us in anticipating that he will continue to improve the subsequent editions, which the demands of the students in the French schools can scarcely fail to call for.

